# Association Between Blood Pressure, Glomerular Filtration Rate, and Serum Thyroid-Stimulating Hormone Levels in Hypothyroid Patients: A Retrospective Single-Center Study

**DOI:** 10.7759/cureus.28686

**Published:** 2022-09-02

**Authors:** Manal O Alsulami, Nada M Alharbi, Dania W Alsulami, Sahar J Almaghrabi, Hadeel A Albaradei, Amani M Alhozali

**Affiliations:** 1 College of Medicine, King Abdulaziz University, Jeddah, SAU; 2 Endocrinology, King Abdulaziz University Hospital, Jeddah, SAU

**Keywords:** creatinine, tsh, hypothyroidism, gfr, bp

## Abstract

Background: Thyroid hormones have substantial effects on blood pressure (BP) and renal function as they influence the glomerular filtration rate (GFR). Maintaining healthy BP and preventing premature development of nephropathy necessitates taking steps.

Objectives: The aim of this study was to explore the association between BP, GFR, and thyroid-stimulating hormone (TSH) levels in hypothyroid patients at King Abdulaziz University Hospital, Jeddah, Saudi Arabia.

Methods: A retrospective record review study of all hypothyroid patients from June 1, 2010 to June 6, 2020. The medical records of 1,181 adult patients were reviewed, and 157 met the criteria. All patients aged >18 years who were diagnosed with hypothyroidism and were on levothyroxine therapy, were included in this study.

Results: More than half of the participants were female (83.4%). There was no significant correlation between TSH and systolic BP (P= 0.6), or TSH and diastolic BP (P=0.8), while there was a positive correlation between TSH and creatinine (r=0.4, P=0.001) and a negative correlation between TSH and GFR (r=−0.2, P=0.01).

Conclusions: We found no association between BP and TSH, while creatinine correlated directly and GFR inversely with TSH. Follow-up renal function should be a target for physicians in hypothyroid patients to prevent premature complications.

## Introduction

Thyroid hormones have substantial effects on blood pressure (BP), lipids, and energy consumption [1]. They affect both pre-renal and direct-renal functions as they influence the cardiovascular system, kidney blood flow, and glomerular filtration rate (GFR) [2].

Hypothyroidism is a common disease. Overt hypothyroidism is defined as serum thyroid-stimulating hormone (TSH) concentration above, and free thyroxine (FT4) below, the normal reference range [3]. A retrospective single-center study conducted in Jeddah, Saudi Arabia, showed that the prevalence of hypothyroidism was 29.1% [4].

Numerous studies have investigated the correlation between TSH levels and BP. In 2019, a study demonstrated that thyroid disorders, including hypothyroidism, can increase the risk of hypertension [5]. Another study showed that elevated systolic BP (SBP) and diastolic BP (DBP) are usually observed in overt hypothyroidism as a result of cardiovascular hemodynamics and functional changes [6].

Additionally, long-standing hypothyroidism can cause significant changes in renal function, serum creatinine levels are elevated, and GFR values are reversibly reduced in overt hypothyroid patients compared to euthyroid subjects [7].

Maintaining healthy BP is a major goal for all individuals, and preventing premature development of nephropathy necessitates intervention. The aim of this study was to explore the association between BP, GFR, and TSH in hypothyroid patients at King Abdulaziz University Hospital (KAUH), in Jeddah, Saudi Arabia.

## Materials and methods

This was a retrospective record review study of all hypothyroid patients attending KAUH from June 1, 2010 to June 6, 2020. The medical records of 1,181 adult patients were reviewed, and 157 met the criteria. Ethical approval was obtained from the Unit of Biomedical Research Ethics Committee, KAUH, College of Medicine (303-20).

Study population and data collection 

Patients aged >18 years who were diagnosed with hypothyroidism and were on levothyroxine therapy were included in this study. We excluded patients who were known to have secondary hypothyroidism or other endocrine disorders such as Cushing’s syndrome, and acromegaly; patients on treatment with drugs that are known to influence renal function, such as amiodarone, lithium, angiotensin-converting enzyme inhibitors/angiotensin receptor blockers, diuretics, allopurinol, steroids, phenytoin, carbamazepine, salicylates, beta-blockers, rifampicin, or cytotoxic drugs; patients with renal disorders, liver disorders, diabetes, hypertension, other autoimmune disorders such as rheumatoid arthritis and systemic lupus erythematosus, or malignancy; and pregnant women.

Data were extracted during July 2020 from the electronic medical record system by using a data collection sheet that collected demographic data including age, sex, nationality, height, weight, BP, TSH, FT4, free triiodothyronine (FT3), and creatinine laboratory test results. A BP < 120/80 mmHg was considered to be normal [8]. The reference ranges for TSH, FT4, FT3, and creatinine, according to the KAUH laboratory are listed in Table 1.

**Table 1:** Laboratory tests and their reference ranges TSH: Thyroid stimulating hormone; FT4: Free thyroxine; FT3: Free triiodothyronine

Laboratory test	Reference range
TSH	0.27–4.2 µIU/L
FT4	2.8–7 pmol/L
FT3	12–22 pmol/L
creatinine	53–115 µmol/L

Body mass index (BMI) was calculated as weight in kilograms divided by height in meters squared (kg/m^2^). BMI was categorized based on the World Health Organization’s cut-offs: underweight (<18.5 kg/m^2^), healthy weight (18.5-24.99 kg/m^2^), overweight (25.0-29.99 kg/m2) or obese (≥30.0 kg/m^2^) [9].

The estimated GFR was calculated using the four-variable Modification of Diet in Renal Disease (MDRD) Study Equation [10]:

GFR = 175 × (sCr − 1.154) × (age − 0.203) × (0.742 if female)

where sCr is serum creatinine in mg/dL. 

Power calculation was based on estimations of differences in GFR in an adult population with and without hypothyroidism [2]. With alpha = 0.05 (two-sided test) and a power of 80%, we would detect a significant difference in hypertension prevalence with a sample size of at least 100 participants.

Statistical analysis

Microsoft Excel 2019 was used for data entry, and statistical analysis was performed using IBMSPSS Statistics version 21 (IBM Corp., Armonk, NY, USA). Continuous variables were reported using means and standard deviations, while categorical variables were reported using frequencies and percentages. Differences between TSH, F4 and F3 levels between anthropometric and demographic groups were analyzed using students' t-test or one-way ANOVA. Correlations between continuous data were analyzed using Pearson’s correlation. P-values < 0.05 were considered statistically significant.

## Results

A total of 157 patients with hypothyroidism were included in the study, of whom 131 (83.4%) were female, and 110 (70.1%) were Saudi nationals. Their mean age was 45 ± 15 years, and their mean BMI was 32.1 ± 19.5 kg/m^2^ (Table 2). 

**Table 2:** Anthropometric and demographic data of study participants BMI: Body mass index; SD: Standard deviation

Features		Result
Age (years) (mean ± SD)		45 ± 14
Sex (n, n%)	Female	131 (83.4%)
	Male	26 (16.6%)
Nationality (n, n%)	National	110 (70.1%)
	Non-national	47 (29.9%)
BMI (kg/m^2^) (mean ± SD)		19.5 ± 2.1

The data in Table 3 show that there was no significant correlation between TSH and SBP (P = 0.6) or TSH and DBP (P = 0.8), while there was a weak positive correlation between TSH and creatinine (r = 0.4, P = 0.001) and a weak negative correlation between TSH and GFR (r = −0.2, P = 0.01). We looked for possible associations between FT4 and SBP, creatinine, and GFR using Pearson correlation analysis showed that FT4 correlated positively with SBP and GFR (r = 0.2, P = 0.003 and r = 0.2, P = 0.02), respectively, although it correlated negatively with creatinine (r = −0.2, P = 0.02). Moreover, it showed that FT3 correlated positively with SBP (r = 0.2, P= 0.005)

**Table 3:** Relationship between thyroid-stimulating hormone, free thyroxine, and free triiodothyronine levels and different clinical and biochemical factors SBP, systolic blood pressure; DBP, diastolic blood pressure; GFR, glomerular filtration rate; TSH, thyroid stimulating hormone; FT4, free thyroxine; FT3, free triiodothyronine

Summary of investigations						
Parameters	TSH (µIU/L)		FT4 (pmol/L)		FT3 (pmol/L)	
	r	p	r	p	r	p
SBP (mmHg)	-0.04	0.6	0.2	0.003	0.2	0.005
DBP (mmHg)	0.01	0.8	0.1	0.4	0.004	0.9
creatinine (µmol/L)	0.4	<0.001	-0.2	0.02	-0.1	0.07
GFR (mL/min/1.73 m^2^)	-0.2	0.01	0.2	0.02	0.2	0.07

Table 4 showed that Creatinine and GFR significantly differed among TSH groups (P = 0.001 and P =0.009 respectively. Creatinine level was highest in patients with TSH >10 µIU/L (82.9 ± 26.1 µmol/L) and this was significantly higher than those with TSH 2.01-4.20 µIU/L who had creatinine 56.1 ± 14.7 µmol/L (p = 0.002). In addition, this patient had the lowest GFR with 73 ± 24 (mL/min/1.73 m^2^). This was significantly lower than patients with TSH 2.01-4.20 µIU/L who had GFR 123.9 ± 49.8 24 (mL/min/1.73 m^2^) (p = 0.019).

**Table 4:** Systolic blood pressure, diastolic blood pressure, serum creatinine and glomerular filtration rate according to the serum thyroid-stimulating hormone level (µIU/L) TSH: thyroid-stimulating hormone; SBP: systolic blood pressure; DBP: diastolic blood pressure; GFR: glomerular filtration rate ^$^Data are shown as mean ± SD. a is significantly different from b (P < 0.05, one-way ANOVA test) ^^^Data are shown as number and percentages. (P-value, chi-square test)

Summary of investigations						
Parameters	TSH (µIU/L)					P-value
	<0.27	0.271–2.00	2.01–4.20	4.21–9.99	≥10	
SBP (mmHg)^$^	127.8 ± 27.9	127.4 ± 16.9	126.9 ± 15.2	125.4 ± 15.4	122.5 ± 12.8	0.92
DBP(mmHg) ^$^	68.3 ± 11.1	75.8 ± 12.5	75.8 ± 9.5	78.2 ± 12.4	72.9 ± 7.2	0.084
creatinine (µmol/L) ^$^	66.8 ± 21.7 ^a,b^	65 ± 20.5 ^a,b^	56.1 ± 14.7 ^a^	63.3 ± 14 ^a,b^	82.9 ± 26.1 ^b^	0.001
GFR(mL/min/1.73m^2^) ^$^	97.3 ± 33.1^ a,b^	110.9 ± 44.7 ^a,b^	123.9 ± 49.8 ^b^	108.2 ± 32.4 ^a,b^	73 ± 24 ^a^	0.009

## Discussion

In this retrospective record review, 131 (83.4%) of the participants were women. Similar results were reported by Aldossari et al. (84.5%) and Aljabri et al. (77.2%) [4,9]. This could be because the female-to-male ratio in hypothyroidism was 5.4:1 [11]. In our sample, 56.7% were obese. A study conducted by Chaudhury et al. found that patients with hypothyroidism (the case group) had a higher BMI than those in the control group. This alteration in BMI could be a reflection of decreased basal metabolic rate and direct TSH stimulation of preadipocyte differentiation, resulting in adipogenesis [12].

Our study did not show an association between BP and TSH, which is similar to studies done by Walsh et al. and De Pergola et al. [13,14]. This is contrary to other previous studies that have demonstrated a significant association [15-17]. The differences in iodine intake around the world may affect the outcome of these studies and may explain this discrepancy [15]. A study done on Danish adults demonstrated that median serum TSH concentration was 16% higher after four years of mandatory salt iodization in two regions with mild or moderate iodine deficiency [18]. A prolonged excess of iodine intake reduces pituitary type-2-deiodinase activity subsequently leading to increasing serum TSH concentration [19]. Additionally, various studies of people with overt or subclinical hypothyroidism have found that after T4 treatment, vascular resistance and arterial stiffness may be reduced [20-23]. It has also been reported that FT3 may contribute directly and indirectly to vasodilation of vascular smooth muscle cells [24].

As in previous studies, TSH was directly correlated with creatinine and inversely correlated with GFR [25,26]. Moreover, FT4 was negatively correlated with creatinine and positively correlated with GFR. A study on the relationship between thyroid dysfunction and serum creatinine found that among 191 hypothyroid patients, serum creatinine was negatively correlated with serum thyroxine (T4) (P<0.001) [27]. This may be because GFR is correlated with cardiac output and circulating blood volume, and this may be decreased due to impaired activity of the renin-angiotensin-aldosterone system in hypothyroid patients [25]. Furthermore, hypothyroidism may also increase creatinine release from muscles [28].

This study had some limitations. First, we took one reading for each SBP and DBP because of a lack of documentation. Second, the generalizability may be limited because the study was conducted in only one healthcare center. One of the strengths of this study is that we excluded patients who were taking medications that might affect the thyroid hormones.

## Conclusions

We found no association between BP and TSH, while TSH was correlated directly with creatinine and inversely with GFR. Physicians should monitor renal function in hypothyroid patients to prevent premature complications. Prospective population-based studies should be conducted to assess whether these associations are sufficiently strong to influence the future risk of nephropathy and to provide an accurate estimate of the association between BP and TSH. A multi-center study with large sample size is warranted.
